# Exploring Virus Diversity in the *Potato leafhopper* (*Empoasca fabae*), an Economically Important Agricultural Pest

**DOI:** 10.3390/v16081305

**Published:** 2024-08-16

**Authors:** Thanuja Thekke-Veetil, Doris Lagos-Kutz, Leslie L. Domier, Nancy K. McCoppin, Glen L. Hartman, Steven J. Clough

**Affiliations:** Soybean/Maize Germplasm, Pathology, and Genetics Research Unit, United States Department of Agriculture—Agricultural Research Service, Urbana, IL 61801, USAdlagos@illinois.edu (D.L.-K.); nancy.mccoppin@usda.gov (N.K.M.); ghartman@illinois.edu (G.L.H.)

**Keywords:** potato leafhopper, *Empoasca fabae*, viral metatranscriptomics, vector-enabled transcriptomics, insect viruses, virus diversity

## Abstract

The potato leafhopper (*Empoasca fabae*, PLH) is a serious pest that feeds on a wide range of agricultural crops and is found throughout the United States but is not known to be a vector for plant-infecting viruses. We probed the diversity of virus sequences in field populations of PLH collected from four Midwestern states: Illinois, Indiana, Iowa, and Minnesota. High-throughput sequencing data from total RNAs extracted from PLH were used to assemble sequences of fifteen positive-stranded RNA viruses, two negative-stranded RNA viruses, and one DNA virus. These sequences included ten previously described plant viruses and eight putative insect-infecting viruses. All but one of the insect-specific viruses were novel and included three solemoviruses, one iflavirus, one phenuivirus, one lispivirus, and one ambidensovirus. Detailed analyses of the novel genome sequences and their evolutionary relationships with related family members were conducted. Our study revealed a diverse group of plant viruses circulating in the PLH population and discovered novel insect viruses, expanding knowledge on the untapped virus diversity in economically important crop pests. Our findings also highlight the importance of monitoring the emergence and circulation of plant-infecting viruses in agriculturally important arthropod pests.

## 1. Introduction

Insects represent a highly diverse group of organisms that have symbiotic and parasitic relationships with plants and animals. Being an integral dietary component of many animals, insects could potentially act as vehicles of virus transport within the animal and plant kingdoms, or between these kingdoms. There have been considerable advancements in research on insect viruses, but this has mainly been skewed towards vector-borne diseases of animals and focused on blood-feeding insects [1]. With advances in sequencing technologies, novel viruses have been discovered from various organisms and challenging environments. Recently, several metagenomic and metatranscriptomic studies have discovered viruses from other insects, including plant feeders such as aphids, beetles, fruit flies, leafhoppers, grasshoppers, lepidopterans, planthoppers, psyllids, soldier flies, stink bugs, thrips, and white flies [2,3,4,5,6,7,8,9,10,11,12,13,14,15,16,17]. Yet, the viromes of most insect species remain unexplored. 

Leafhoppers are one of the most abundant insect groups belonging to the family *Cicadellidae* under the order Hemiptera. They fall under the suborder Auchenorrhyncha [18] and have broad host ranges. These plant feeders have mouth parts adapted for piercing and sucking sap from a wide range of plants species. Leafhopper feeding damages plants, causing the ‘burning’ of foliage known as ‘hopper burn’, a characteristic symptom of hopper infestation. Potato leafhoppers (PLHs, *Empoasca fabae*) are native to North America and are a common and serious pest in the United States, affecting over 100 species of host plants [19]. Potato leafhoppers are migratory pests in the Midwest as they cannot survive the cold winter. During the winter, PLHs migrate and reproduce on the Gulf Coast, and in the spring the winged adults are caught in wind currents and transported back. If PLHs arrive early in the growing season, they feed and survive on the alternate weed hosts in the field and subsequently infest crops once they are established. In late summer when the crops senesce, PLHs move to native plants again and feed until they migrate south for the winter [20].

Some species of leafhoppers can transmit pathogens associated with plant sap, such as bacteria, phytoplasma, and viruses [21,22,23]. More than 50 species of leafhoppers under 25 genera are reported to be vectors of plant viruses, including badnaviruses, geminiviruses, phytoreoviruses, picornaviruses, rhabdoviruses, and waikaviruses [22,24,25,26,27,28,29]. The mechanisms of virus transmission by leafhoppers vary depending on the species of leafhopper and the virus involved. Modes of virus transmission by leafhoppers include semipersistent transmission of foregut-borne viruses, persistent transmission of circulative viruses, and persistent transmission of propagative viruses [22]. 

Although symptoms induced by PLH resemble those induced by plant viruses, studies have failed to demonstrate the ability of PLH to transmit plant-infecting viruses [23,30]. Our communication is the first report on a broad range of viruses detected in PLH. Also, our study expands knowledge on the diverse viruses present in arthropod pests of common agricultural crops. 

## 2. Materials and Methods

### 2.1. Potato Leafhopper Collection and Sample Preparation for High-Throughput Sequencing

Leafhoppers were collected using the Midwest Suction Trap Network [31] installed in corn and soybean fields in Illinois (Freeport, Morris, Orr, and Urbana-Champaign); Iowa (Ames, Kanawha, Nashua, and Sutherland); Indiana (Wanatah); and Minnesota (Crookston, Lamberton, Morris, and Rosemount). Among the insects collected in the suction traps, hoppers were separated and PLHs were identified based on the morphological characteristics described [32]. Potato leafhoppers were then stored in 95% ethanol at −20 °C until processed. Samples from Illinois were collected in the 2020 and 2021 growing seasons; samples from Iowa and Indiana were collected in 2020; and samples from Minnesota were collected in 2021. A total of 593 individuals were collected from suction traps in Illinois (480), Indiana (13), Iowa (57), and Minnesota (43). For high-throughput sequencing, 13 (Indiana) to 15 (Iowa and Minnesota) randomly selected individuals were pooled into one sample for each state. Due to the large number of PLHs collected from Illinois, a total of 137 individuals (107 for 2020 and 30 for 2021) were used for total RNA extractions. Total RNA was extracted using the RNeasy Mini Kit (Qiagen, Valentia, CA, USA), treated with Turbo DNase (ThermoFisher, Waltham, MA, USA), and depleted of ribosomal RNA (rRNA) with the Illumina Ribo-Zero Plus rRNA Depletion Kit (Illumina, San Diego, CA, USA). A total of five sequencing libraries were prepared separately for each state and each year (libraries for IL, IA and IN for 2020 and libraries for IL and MN for 2021) using the Illumina ScriptSeq RNA-Seq Library Preparation Kit and sequenced on an Illumina NovaSeq 6000 at the Roy J. Carver Biotechnology Center at the University of Illinois as 100 nt paired-end reads. The sequence libraries produced from 7.71 × 10^7^ to 1.62 × 10^8^ paired-end reads each (Table 1).

### 2.2. Assembly of Virus-Like Sequences from the Leafhopper Transcriptome

Sequence reads from each library were trimmed using Trimmomatic [33] and de novo assembled with Trinity [34]. Contigs were compared to a sequence database that included reference invertebrate and virus amino acid (aa) sequences (NCBI GenBank Release 236) using USEARCH [35]. Contigs with significant similarity to virus sequences were compared to the NCBI nonredundant protein database using BLASTX [36]. The number of reads aligning to each virus sequence and depths of coverage were calculated using Bowtie2 [37] and SAMtools [38]. The raw reads were deposited in the SRA database under the Bioproject accession PRJNA802548. The GenBank accession numbers of the virus genomes assembled from the PLH transcriptome data are presented in Table 2. Sequence data are presented only for contigs larger than 500 nucleotides (nt). The open reading frames (ORFs) of virus genomes were identified using ORF finder [39] and the predicted sizes of the encoded proteins were estimated using Protein Molecular Weight [40]. For phylogenetic analyses, RNA-dependent RNA polymerase (RdRp) sequences of novel viruses and taxonomically related members were aligned using the CLUSTALW program in MEGA 11 [41], and maximum likelihood trees were constructed with 500 bootstrap replications.

## 3. Results

The transcriptome data of PLH collected from the Midwestern states of Illinois, Iowa, Indiana, and Minnesota contained both plant- and insect-specific virus sequences. The details of the sequence reads obtained from the five sequencing libraries are presented in Table 1. 

**Table 1:** Numbers of sequence reads for viruses detected in potato leafhoppers’ RNA-seq data.

	Numbers of Aligned Sequence Reads				
Plant Viruses	IL 2020 ^1^ (*n* = 107) ^2^	IL 2021 (*n* = 30)	IA 2020 (*n* = 15)	IN 2020 (*n* = 13)	MN 2021 (*n* = 15)
Barley yellow dwarf virus-PAV NC_002160.2	0	0	0	19	0
Clover yellow mosaic virus NC_001753.1	13	0	4	0	0
Lucerne transient streak virus NC_001696.2	522	0	4	0	0
Peanut stunt virus RNA2 NC_002039.1	18	0	0	0	0
Peanut stunt virus RNA1 NC_002038.1	19	0	0	0	0
Peanut stunt virus RNA3 NC_002040.1	17	0	0	0	0
Red clover vein mosaic virus NC_012210.1	138	0	8	0	0
Red clover necrotic mosaic virus RNA1 NC_003756.1	0	0	220	0	0
Red clover necrotic mosaic virus RNA2 NC_003775.1	0	0	34	0	0
Soybean carlavirus 1	5262	0	0	0	0
Turnip vein-clearing virus NC_001873.1	130	8	0	25	0
White clover mosaic virus NC_003820.1	86	0	3	1	0
White clover mottle virus NC_031747.1	852	0	0	37	0
Insect Viruses					
Empoasca fabae solemovirus 1	3498 [3000 nt] ^3^	9149 [3022 nt]	1811 [2999 nt]	0 NC ^4^	0 NC
Empoasca fabae solemovirus 2	24,633 [2935 nt]	151,541 [2941 nt]	7 NC	44 [1734 nt]	132,567 [2957 nt]
Empoasca fabae solemovirus 3	116,809 [2820 nt]	35,666 [2815 nt]	330,243 [3025 nt]	132,419 [2818 nt]	53,827 [2818 nt]
Marma virus RNA1 OM817544.1	0 NC	80 [858 nt]	9 NC	0 NC	7 NC
Marma virus RNA2 OM817545.1	0 NC	46 [1650 nt]	2 NC	0 NC	0 NC
Empoasca fabae iflavirus 1	44,850 [10,785 nt]	192,880 [10,857 nt]	111,614 [10,877 nt]	208,341 [10,854 nt]	40,699 NC
Empoasca fabae lispivirus 1	49,575 [14,523 nt]	0 NC	82 NC	0 NC	298 [1380 nt]
Empoasca fabae phenuivirus	165,436 [9330 nt]	125,122 [9325 nt]	423,949 [9389 nt]	212,198 [9350 nt]	0 NC
Empoasca fabae densovirus 1	586 [3837 nt]	303 [1689 nt]	661 [3676 nt]	480 [2242 nt]	598 NC
Total sequence reads in library	7.71 × 10^7^	1.29 × 10^8^	1.62 × 10^8^	1.31 × 10^8^	1.31 × 10^8^

^1^ Location and year of collection of leafhopper samples for RNA-seq library preparation. ^2^ Numbers of potato leafhopper individuals used in the preparation of RNA-seq libraries. ^3^ Length of longest contig assembled for each virus from each library. ^4^ No contig (NC) of greater than 500 nt was assembled for the designated virus for the location and year.

### 3.1. Plant Viruses Discovered from PLH

Sequences from 10 previously identified positive-strand RNA plant-infecting viruses were identified from PLH RNA-seq data (Table 2). These viruses were barley yellow dwarf virus (BYDV)-PAV, clover yellow mosaic virus (ClYMV), lucerne transient streak virus (LTSV), peanut stunt virus (PSV), red clover vein mosaic virus (RCVMV), red clover necrotic mosaic virus (RCNMV), soybean carlavirus 1 (SCV-1), turnip vein-clearing virus (TVCV), white clover mosaic virus (WClMV), and white clover mottle virus (WCMoV). These viruses are either seed-borne, transmitted mechanically, or transmitted by aphids or beetles, but none have been reported to be transmitted by leafhoppers [42,43,44,45,46,47].

Among the plant viruses, nearly complete genomes were assembled for RCNMV and SCV-1 (Table 2). The PLH RCNMV sequence (accession number, PP946270) contained all coding regions of RNA1 of its bipartite genome, which encoded a 27 kDa replication-associated protein, 86 kDa viral polymerase, and a 36 kDa coat protein (CP). The predicted aa sequences of these proteins were 97.6%, 89.4%, and 79.6% identical, respectively, to the corresponding proteins encoded by the reference RCNMV RNA1 sequence (NC_003756.1).

A nearly complete genome (8223 nt) was assembled from PLH for SCV-1 (accession number, PP946266) (Table 2). Soybean carlavirus 1 was originally identified from RNA-seq data from soybean plants [48] and from soybean thrips [15]. Sequence comparison of the insect-derived (PLH and soybean thrips) SCV-1 isolates revealed very high levels of aa sequence identity: 95.7% in replicase, 98.7% in triple gene block (TGB) 1 protein, 100% in TGB2 and TGB3 proteins, and 99% in CP. Contigs representing more than half the genomes of LTSV (2953 nt, accession number, PP946263) and WCMoV (3366 nt, accession number, PP946269) were assembled from the Illinois 2020 PLH RNA-seq data.

### 3.2. Insect-Specific Virus-Like Sequences Discovered from PLH

**Figure 1:**
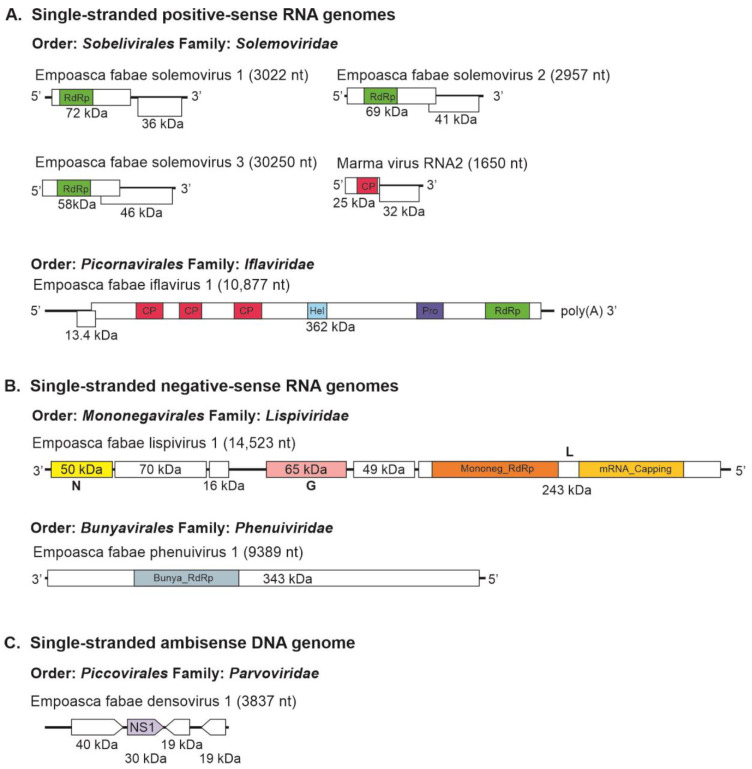
Genome organization of novel insect-specific viruses identified from potato leafhopper (*Empoasca fabae*) transcriptome data with single-stranded positive-sense RNA (**A**), single-stranded negative-sense RNA (**B**), and single-stranded ambisense DNA (**C**) genomes. Boxes represent open reading frames. Colored boxes indicate conserved domains: RdRp = RNA-dependent RNA polymerase; Pro = protease; Hel = helicase; CP = capsid protein; Mononeg_RdRp = *Mononegavirales* RdRp; mRNA_capping = *Mononegavirales* mRNA-capping region; N = bunyavirus nucleocapsid protein; G = bunyavirus glycoprotein; L = bunyavirus replicase; Bunya_RdRp = bunyavirus RNA-dependent RNA polymerase; NS1 = parvovirus nonstructural protein 1 (NS1) domain.

Eight insect-specific virus sequences, representing the genomes of seven novel and one previously reported virus, were assembled from the PLH transcriptome data (Table 2). These included sequences of five positive-strand RNA viruses, two negative-strand RNA viruses, and one DNA virus. Among the positive-strand RNA viruses, four solemovirus genomes were assembled, including one previously described Marma virus. The three novel solemoviruses were tentatively named Empoasca fabae solemovirus (EFSV)1 (independently assembled from three of the five libraries); EFSV-2 (independently assembled from four of the five libraries); and EFSV-3 (independently assembled from all five libraries) (Table 1). The nt sequences of the three isolates of EFSV-1 discovered were 91.2% to 92.6% identical; the four isolates of EFSV-2 were 93.6% to 96.8% identical; and the five isolates of EFSV-3 were 95.1% to 97.1% identical. The genomes of these viruses contained two ORFs, which overlapped in EFSV-2 (accession number, PP946275) and EFSV-3 (accession number, PP946280) but not in EFSV-1 (accession number, PP946277) (Figure 1). The first ORFs of EFSV-1, EFSV-2, and EFSV-3 encoded hypothetical proteins of 72 kDa, 69 kDa, and 58 kDa, respectively. The second ORFs encoded putative replicases of 36 kDa, 41 kDa, and 46 kDa, respectively. 

**Table 2:** Viruses discovered from the transcriptome sequencing of potato leafhoppers collected from four Midwestern states in the United States.

Viruses	Accession Number	Family	Longest Contig	Best Match (Accession Number)	% aa Identity	% Coverage
Plant viruses						
Barley yellow dwarf virus-PAV	PP946271	*Solemoviridae*	467	Barley yellow dwarf virus-PAV (NC_004750.1)	97.9	8.2
Clover yellow mosaic virus	PP946262	*Alphaflexiviridae*	276	Clover yellow mosaic virus (NC_001753.1)	73.2	3.9
Lucerne transient streak virus	PP946263	*Solemoviridae*	2953	Lucerne transient streak virus (NC_001696.2)	97.5	69.0
Peanut stunt virus RNA1	PP946264	*Bromoviridae*	447	Peanut stunt virus RNA1 (NC_002038.1)	96.6	13.3
Red clover vein mosaic virus	PP946265	*Betaflexiviridae*	1010	Red clover vein mosaic virus (NC_012210.1)	96.8	11.7
Red clover necrotic mosaic virus RNA1	PP946270	*Tombusviridae*	3851	Red clover necrotic mosaic virus RNA1 (NC_003756.1)	92.9	99.0
Soybean carlavirus 1	PP946266	*Betaflexiviridae*	8223	Soybean carlavirus 1 (MW349427.1)	96.0	95.1
Turnip vein-clearing virus	PP946267	*Virgaviridae*	1961	Turnip vein-clearing virus (NC_001873.1)	99.4	31.1
White clover mosaic virus	PP946268	*Alphaflexiviridae*	1048	White clover mosaic virus (NC_003820.1)	98.8	17.9
White clover mottle virus	PP946269	*Solemoviridae*	3366	White clover mottle virus (NC_031747.1)	98.9	54.2
Insect viruses						
Empoasca fabae solemovirus 1	PP946277	*Solemoviridae*	3022	Amygdalus persica sobemo-like virus (QKI29237.1)	45.1	92.8
Empoasca fabae solemovirus 2	PP946275	*Solemoviridae*	2957	Amygdalus persica sobemo-like virus (QKI29238.1)	47.8	90.8
Empoasca fabae solemovirus 3	PP946280	*Solemoviridae*	3025	Solemoviridae sp. (QXV86398.1)	50.5	100.2
Marma virus RNA 1	PP946293	*Solemoviridae*	858	Marma virus RNA1 (OM817544.1)	99.8	27.3
Marma virus RNA 2	PP946286	*Solemoviridae*	1650	Marma virus RNA2 (OM817545.1)	99.6	101.5
Empoasca fabae iflavirus 1	PP946291	*Iflaviridae*	10,877	Scaphoideus titanus iflavirus 1 (QIJ56901.1)	46.6	101.4
Empoasca fabae lispivirus 1	PP946292	*Lispiviridae*	14,523	Hemipteran arli-related virus OKIAV95 (QPL15300.1)	40.1	192.8
Empoasca fabae phenuivirus 1	PP946285	*Phenuiviridae*	9330	Blattodean phenui-related virus OKIAV266 (QMP82340.1)	30.5	130.7
Empoasca fabae densovirus 1	PP946287	*Parvoviridae*	3837	Motacilla cinerea parvoviridae sp. (QTE03821.1)	40.0	71.1

**Figure 2:**
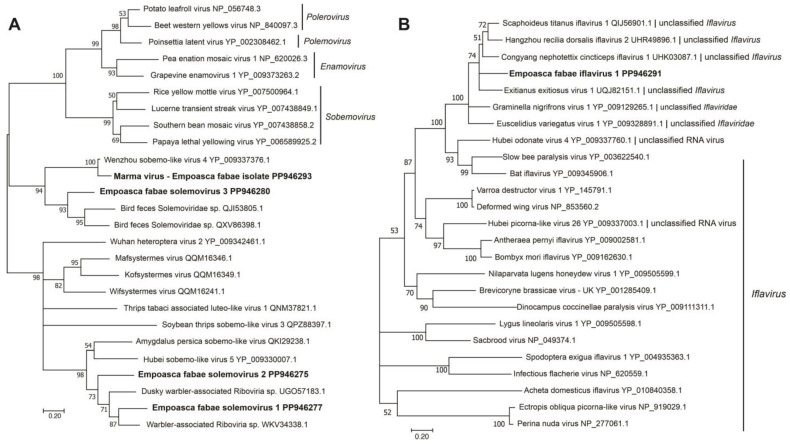
Phylogenetic analysis of predicted RNA-dependent RNA polymerase amino acid sequences encoded by contigs assembled from potato leafhopper (*Empoasca fabae*) transcriptome data and related viruses in the families *Solemoviridae* (**A**) and *Iflaviridae* (**B**). Predicted amino acid sequences containing RNA-dependent RNA polymerase domains were aligned using MUSCLE. Phylogenetic analyses were performed using the maximum likelihood method in MEGA11. Numbers at nodes indicate percent bootstrap support (500 replicates). Nodes with less than 50% bootstrap support were collapsed to the next higher level. GenBank accession numbers and taxonomic designation (where available) are indicated after each sequence name. Sequences identified from potato leafhopper transcriptome data are indicated in bold.

A contig representing a near full-length sequence for RNA2 of Marma virus (accession number, PP946286) was also obtained from the Illinois 2021 PLH samples. Marma virus is an unclassified member of *Solemoviridae* associated with mosquitoes [49]. This sequence contained coding regions of its 25 kDa CP and 32 kDa hypothetical proteins. Five contigs (191 nt, 394 nt, 618 nt, 733, nt, and 858 nt) representing 86% coverage of Marma virus RNA1 were also assembled from the Illinois 2021 RNA-seq data. In phylogenetic analysis, the previously described plant-infecting members of *Solemoviridae* and the unclassified solemo-like viruses reported from arthropods formed two main clades in which the PLH solemovirus sequences branched with arthropod-associated viruses (Figure 2A).

Genome sequences of greater than 10 kb were assembled for a novel iflavirus from four of the five RNA-seq libraries (Table 1 and Table 2) that was tentatively named as Empoasca fabae iflavirus 1 (EFIV-1). The EFIV-1 genome (10,877 nt, accession number, PP946291) contained a small ORF, uORF, upstream of the main ORF capable of encoding a 13.4 kDa protein (Figure 1). The uORF was present in all four EFIV-1 isolate sequences discovered from PLH data. Some other members of the *Picornavirales* have small ORFs upstream of the initiation codon for their primary ORFs [50,51]. In enteroviruses, uORFs have been shown to modulate virus infection in gut epithelial cells [52]. The EFIV-1 primary ORF was predicted to encode a 362 kDa polyprotein with structural protein domains proximal to the amino terminus and nonstructural protein domains proximal to the carboxyl terminus. In phylogenetic analysis, EFIV-1 grouped with bat iflavirus, slow bee paralysis virus, and several iflavirus sequences from arthropods (Figure 2B).

One of the two novel negative-stranded RNA virus genomes discovered from PLH was a lispivirus (accession number, PP946292) tentatively named as Empoasca fabae lispivirus 1 (EFLV-1). A genome of 14.5 kb was assembled from the 49,575 reads obtained from the Illinois 2020 RNA-seq library. Low numbers of sequence reads for the virus were also detected in the Iowa data (82 reads) and the Minnesota (298 reads) data (Table 1). The genome contained six ORFs (Figure 1), which was predicted to encode proteins of 50 kDa, 70 kDa, 16 kDa, 65 kDa, 49 kDa, and 243 kDa. The predicted aa sequences of ORFs 4 and 6 showed similarity to the glycoprotein and RdRp, respectively, of the viruses in Lispiviridae family in order Mononegavirales. 

**Figure 3:**
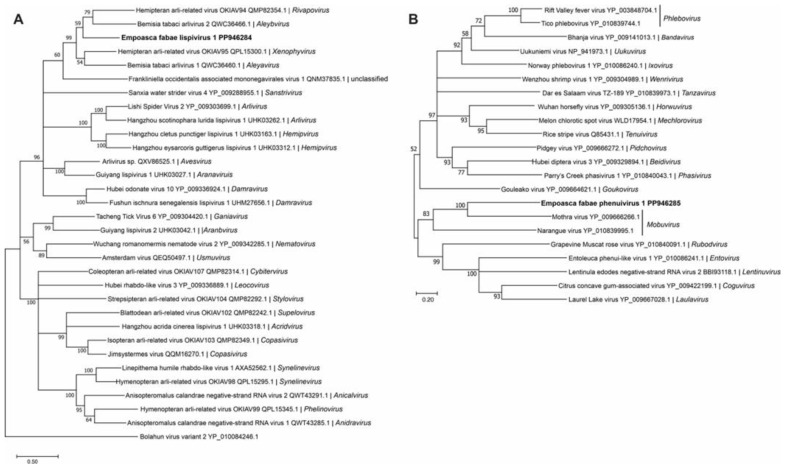
Phylogenetic analysis of predicted RNA-dependent RNA polymerase amino acid sequences encoded by contigs assembled from potato leafhopper (*Empoasca fabae*) transcriptome data and related negative-sense RNA viruses in the families *Lispiviridae* (**A**) and *Phenuiviridae* (**B**). See Figure 2 legend for details.

The predicted amino acid sequence of the EFLV-1 RdRp grouped most closely with members of the *Aleyavirus*, *Aleybvirus*, *Rivapovirus*, and *Xenophyvirus* genera in the family *Lispiviridae* (Figure 3A). An L- segment of greater than 9.3 kb (accession number, PP946285) of a new putative member of the family *Phenuiviridae*, Empoasca fabae phenuivirus 1 (EFPV-1), was assembled from all five libraries (Table 1). The RNA was predicted to encode a 343 kDa RdRp (Figure 1). *Phenuiviridae* members have tripartite negative-stranded RNA genomes with L, M, and S segments. Most are transmitted by arthropod vectors. In phylogenetic analyses, EFPV-1 grouped with Mothra virus and Narangue virus of the genus *Mobuvirus* in the *Phenuiviridae* family (Figure 3B).

**Figure 4:**
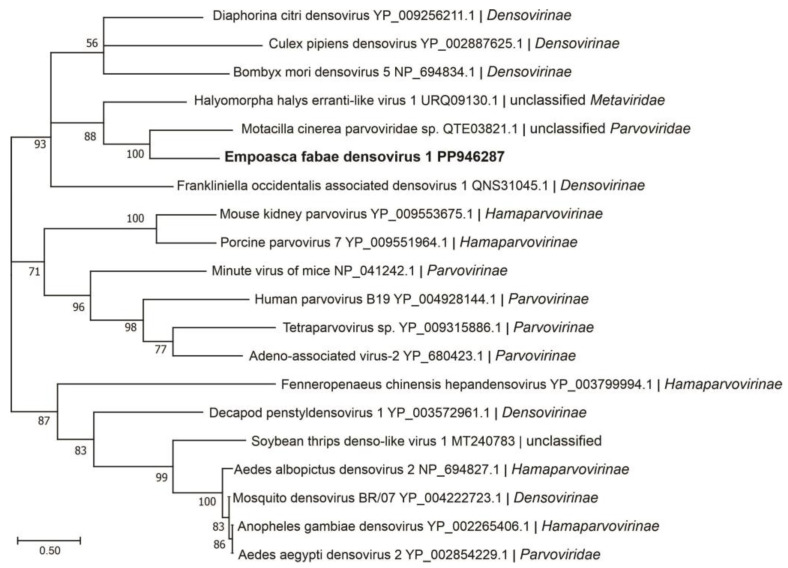
Phylogenetic analysis of predicted NS1 amino acid sequences encoded by contigs assembled from potato leafhopper (*Empoasca fabae*) transcriptome data and related viruses in the family *Parvoviridae*. See Figure 2 legend for details.

Four assembled contigs of a DNA virus resembling the genomes of ambidensoviruses were discovered from the samples of PLH and the virus was provisionally named as Empoasca fabae densovirus 1 (EFDV-1). Densoviruses are small DNA viruses with single-stranded linear genomes of 4–6 kb and are grouped in the *Densovirinae* subfamily of the *Parvoviridae* family. The *Densovirinae* subfamily contains five genera, among which members of the *Ambidensovirus* genus have protein-coding regions on both DNA strands. The nonstructural proteins are encoded in the 5′-proximal of the positive strand, while the structural proteins are encoded in the 5′-proximal of the negative strand [53]. EFDV-1 sequence (3837 nt, accession number, PP946287), contained four ORFs (Figure 1), ORFs 1 and 2 are on the positive strand while ORFs 3 and 4 are on the negative strand, and encoded proteins of 40 kDa, 30 kDa, 19 kDa, and 19 kDa, respectively. Among these proteins, only the ORF2-encoded protein (non-structural protein 1) showed a relationship with members of *Parvoviridae* (Table 2). In phylogenetic analysis, EFDV-1 grouped with members of the *Parvoviridae* family in the subfamily *Densovirinae* (Figure 4).

### 3.3. Virus Distribution in PLH Collected from Midwestern States 

Among the plant viruses detected in PLH data, all except two, BYDV-PAV and RCNMV, were present in PLH collected from Illinois (Table 1). No plant viruses were detected in PLH from Minnesota. Four of the viruses were detected only in a single state: BYDV-PAV only in Indiana; PSV and SCV-1 only in Illinois; and RCNMV only in Iowa. 

Compared to plant viruses, insect-specific virus sequences had much higher read counts and most were detected in all five RNA-seq libraries with a few exceptions. EFSV-1 was not present in PLH collected from Indiana and Minnesota; Marma virus and EFLV-1 were not detected in Indiana; and EFPV-1 was not detected in Minnesota.

## 4. Discussion

Because of their importance for human health, studies of insect viral diversity have been biased towards blood-feeding arthropods. Few studies have reported on viral diversity in agriculturally relevant arthropods that include vectors and non-vectors of plant viruses [2,3,4,5,6,7,8,9,10,11,12,13,14,15,16,17]. Potato leafhoppers are widespread, economically important pests affecting a wide range of crops in the US. Leafhoppers feed on the phloem, xylem, and mesophyll cells of plants [54], and in the process PLH could potentially acquire, harbor, and/or transmit pathogens including viruses. Studies have shown that some species in the genus Empoasca are vectors of plant viruses [55]. An early study described PLH as a non-vector of plant viruses [56], and there have been no subsequent reports of PLH vectoring plant viruses.

We discovered ten previously described plant viruses from PLH transcriptome data (Table 2). Six of these (BYDV-PAV, LTSV, PDSV, RCVMV, SCV-1, and WClMoV) belong to families of viruses transmitted by aphids or beetles and four (ClYMV, RCNMV, TVCV, and WClMV) to families whose members do not have biological vectors. 

In the United States, LTSV was first discovered in soybean thrips [15] and reported naturally infecting alfalfa [57]. White clover mottle virus, an unclassified member of *Solemoviridae*, has not been previously detected in North America. There are no published reports of the occurrence of WCMoV. However, detection of WCMoV in white clover samples from South Korea has been documented in GenBank (accession number, NC031747). Its presence in PLH indicates the existence of WCMoV in either crop or weed species in North America. Because of their migratory nature and extensive host range, plant viruses discovered from PLH could possibly include those that were acquired from crop and weed plants, locally as well as from their overwintering regions. Potato leafhoppers have not been reported to transmit the plant viruses detected in our study or any other plant viruses. Also, it is interesting to note that no known economically important viruses of the major host plants of PLH in the US were detected in the RNA-seq data. These observations could indicate that, while PLHs can acquire plant viruses, they are unable to transmit viruses to host plants. As illustrated by the detection of plant-infecting viruses not previously reported in North America from leafhoppers, monitoring the viromes of polyphagous insect pests like PLH may provide insights into the populations of plant-infecting viruses circulating in a geographic region that would otherwise be difficult to achieve without sampling a large number of plant species.

Two promising uses of insect viruses are their use as gene delivery and expression vectors in insects and their use as biological control agents. Further studies are needed to determine the distribution of the novel viruses in the field populations of PLH, the impact of these viruses on their physiology and survival, and their potential uses in biological studies and as control agents for PLH management.

Our study discovered eight insect-specific viruses, seven novel viruses, and a mosquito-associated Marma virus [49] from PLHs. Potato leafhoppers could have acquired Marma virus directly from plants on which Marma virus-infected mosquitoes had fed or, like some other invertebrate viruses, Marma virus may have a broad host range that includes both PLH and mosquitoes. The absence of certain viruses in certain states under study could be due to the small sizes of PLH populations analyzed in those states or differences in circulating viruses in the PLH population in those areas. Additional experiments will be required to determine whether these viruses can infect/replicate in PLH or if they were merely just acquired during PLH feeding. 

## 5. Conclusions

Viruses are abundant microorganisms that have the potential to parasitize any living organism. Diversity in many agricultural virus pests is unexplored. Through metatranscriptome analysis, we discovered a broad range of viruses, including novel arthropod viruses and existing plant viruses in PLH for the first time (Supplementary Materials).

## Data Availability

The raw sequence reads used in this study were deposited in the SRA database under the Bioproject accession PRJNA802548.

## Supplementary Materials

The following supporting information can be downloaded at: https://www.mdpi.com/article/10.3390/v16081305/s1. Virus sequences generated and analyzed in this study: Supplemantary_data_1.xlsx.
